# Swarm Robotic Behaviors and Current Applications

**DOI:** 10.3389/frobt.2020.00036

**Published:** 2020-04-02

**Authors:** Melanie Schranz, Martina Umlauft, Micha Sende, Wilfried Elmenreich

**Affiliations:** ^1^Lakeside Labs GmbH, Klagenfurt, Austria; ^2^Institute of Networked and Embedded Systems, University of Klagenfurt, Klagenfurt, Austria

**Keywords:** swarm intelligence, swarm robotics, swarm behavior, swarm robotic applications, cyber-physical systems

## Abstract

In swarm robotics multiple robots collectively solve problems by forming advantageous structures and behaviors similar to the ones observed in natural systems, such as swarms of bees, birds, or fish. However, the step to industrial applications has not yet been made successfully. Literature is light on real-world swarm applications that apply actual swarm algorithms. Typically, only parts of swarm algorithms are used which we refer to as basic swarm behaviors. In this paper we collect and categorize these behaviors into spatial organization, navigation, decision making, and miscellaneous. This taxonomy is then applied to categorize a number of existing swarm robotic applications from research and industrial domains. Along with the classification, we give a comprehensive overview of research platforms that can be used for testing and evaluating swarm behavior, systems that are already on the market, and projects that target a specific market. Results from this survey show that swarm robotic applications are still rare today. Many industrial projects still rely on centralized control, and even though a solution with multiple robots is employed, the principal idea of swarm robotics of distributed decision making is neglected. We identified mainly following reasons: First of all, swarm behavior emerging from local interactions is hard to predict and a proof of its eligibility for applications in an industrial context is difficult to provide. Second, current communication architectures often do not match requirements for swarm communication, which often leads to a system with a centralized communication infrastructure. Finally, testing swarms for real industrial applications is an issue, since deployment in a productive environment is typically too risky and simulations of a target system may not be sufficiently accurate. In contrast, the research platforms present a means for transforming swarm robotics solutions from theory to prototype industrial systems.

## 1. Introduction

Swarms typically consist of many individual, simple, and homogeneous or heterogeneous agents (Dorigo and Birattari, 2007). They traditionally cooperate without any central control, and act according to simple and local behavior. Only through their interactions a collective behavior emerges that is able to solve complex tasks. These characteristics lead to the main advantages of swarms: adaptability, robustness, and scalability. Swarms can be considered as a kind of quasi-organism that can adapt to changes in the environment by following specific behaviors (Hamann and Schmickl, 2012), e.g.:

- Pursuing a specific goal.
- Aggregating or dispersing in the environment.
- Communicating (direct, indirect).
- Memorizing (local states, morphologies).

In swarm robotics, multiple robots—homogeneous or heterogeneous—are interconnected, forming a swarm of robots. Since individual robots have processing, communication and sensing capabilities locally on-board they are able to interact with each other, and react to the environment autonomously.

In this paper, we focus on swarm intelligence applied in the swarm robotics domain. The theoretical and mathematical foundations of traditional swarm algorithms are out of the scope of this paper, as this was already done by multiple other authors at a greater level of detail. For example, Bonabeau et al. (1999) depict phenomena in social insects that had been transferred successfully to algorithms. Biological swarm behaviors from which a number of computational algorithms were developed are also discussed by Parpinelli and Lopes (2011). Camazine et al. (2001) discuss general self-organization aspects in biological systems. Moreover, Garnier et al. (2007) provide a good overview of the biological principles of swarm intelligence. Floreano and Mattiussi (2008) discuss swarm intelligence alongside evolutionary computation, artificial neural networks, and bio robotics. Blum and Li (2008), Binitha and Sathya (2012), and Krause et al. (2013) address swarm intelligence algorithms for optimization. Hassanien and Alamry (2015) depict the natural inspirations of swarm intelligence–based optimization algorithms. Swarm intelligence–based optimization algorithms are analyzed by Yang et al. (2013), the link between swarm intelligence–based optimization algorithms and self-organization is examined by Yang et al. (2017). Rossi et al. (2018) classify existing multi-agent algorithms according to their underlying mathematical structure.

Despite the large number of swarm algorithms, the step to industrial applications has not been mastered successfully, yet. In our research work on real-world applications, we noticed that oftentimes industry applications use the term “swarm,” but typically do not implement particular swarm algorithms. They rather use parts of swarm algorithms and implement them using centralized control. We refer to such parts of swarm algorithms as basic swarm behaviors in the following.

The rest of the paper is organized as follows: In section 2 we propose a taxonomy of basic swarm behaviors. In section 3 we show where these behaviors are applied by giving a comprehensive overview on current swarm robotics research platforms, projects, and products. This overview is complemented by a discussion that analyses the current situation, and explores open challenges in swarm robotics applications. Finally, in section 4 we conclude the paper.

## 2. Basic Swarm Behaviors for Swarm Robotics

In most swarm algorithms, individuals perform according to local rules and the overall behavior emerges organically from the interplay of the individuals of the swarm. Translated to the swarm robotics domain, individual robots exhibit a behavior that is based on a local rule set which can range from a simple reactive mapping between sensor inputs and actuator outputs to elaborate local algorithms. Typically, these local behaviors incorporate interactions with the physical world, including the environment and other robots (Floreano and Mattiussi, 2008). Each interaction consists of reading and interpreting the sensory data, processing this data, and driving the actuators accordingly. Such a sequence of interactions is defined as basic behavior that is repeatedly executed, either indefinitely or until a desired state is reached.

In the following subsections, we classify and list the basic swarm behaviors which are adapted and expanded from Brambilla et al. (2013) with additional swarm robotic behaviors including collective localization, collective perception, synchronization, self-healing, and self-reproduction. The behaviors are explained from a high level view describing the task of individual robots and the resulting global objective achieved by the swarm. We do not detail on the sensing and actuation part which is specific to each robotic platform.

### 2.1. Taxonomy

**Figure 1:**
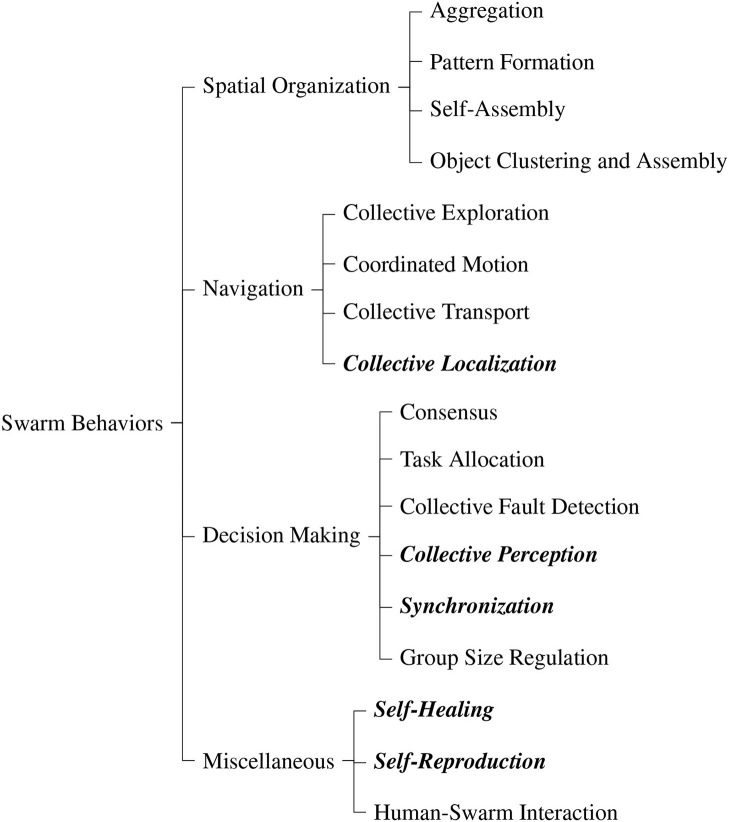
Taxonomy of swarm behaviors adapted from Brambilla et al. (2013). The highlighted behaviors are newly added.

The taxonomy of swarm behaviors is given in Figure 1. It is based on the classification by Brambilla et al. (2013) which we extended by several categories. In the following, we first give an overview of the taxonomy and then an in-detail description of the additional behavior categories in subsection 2.2. For these behaviors we also give the original inspiration. For a detailed description of the existing categories we kindly refer the reader to Brambilla et al. (2013).

#### 2.1.1. Spatial Organization

These behaviors allow the movement of the robots in a swarm in the environment in order to spatially organize themselves or objects.

- **Aggregation** moves the individual robots to congregate spatially in a specific region of the environment. This allows individuals of the swarm to get spatially close to each other for further interaction.
- **Pattern formation** arranges the swarm of robots in a specific shape. A special case is chain formation where robots form a line, typically to establish multi-hop communication between two points.
- **Self-assembly** connects the robots in order to establish structures. They can either be connected physically or virtually through communication links. A special case is morphogenesis where the swarm evolves into a predefined shape.
- **Object clustering and assembly** lets the swarm of robots manipulate spatially distributed objects. Clustering and assembling of objects is essential for construction processes.

#### 2.1.2. Navigation

These behaviors allow the coordinated movement of a swarm of robots in the environment.

- **Collective exploration** navigates the swarm of robots cooperatively through the environment in order to explore it. It can be used to get a situational overview, search for objects, monitor the environment, or establish a communication network.
- **Coordinated motion** moves the swarm of robots in a formation. The formation can have a well-defined shape, e.g., a line, or be arbitrary as in flocking.
- **Collective transport** by the swarm of robots enables to collectively move objects which are too heavy or too large for individual robots.
- ***Collective localization*** allows the robots in the swarm to find their position and orientation relative to each other via establishment of a local coordinate system throughout the swarm. See section 2.2 for more details.

#### 2.1.3. Decision Making

These behaviors allow the robots in a swarm to take a common choice on a given issue.

- **Consensus** allows the individual robots in the swarm to agree on or converge toward a single common choice from several alternatives.
- **Task allocation** assigns arising tasks dynamically to the individual robots of the swarm. Its goal is to maximize performance of the entire swarm system. If the robots have heterogeneous capabilities, the tasks can be distributed accordingly to further increase the system's performance.
- **Collective fault detection** within the swarm of robots determines deficiencies of individual robots. It allows to determine robots that deviate from the desired behavior of the swarm, e.g., due to hardware failures.
- ***Collective perception*** combines the data locally sensed by the robots in the swarm into a big picture. It allows the swarm to make collective decisions in an informed way, e.g., to classify objects reliably, allocate an appropriate fraction of robots to a specific task, or to determine the optimal solution to a global problem. See section 2.2 for more details.
- ***Synchronization*** aligns frequency and phase of oscillators of the robots in the swarm. Thereby, the robots have a common understanding of time which allows them to perform actions synchronously. See section 2.2 for more details.
- **Group size regulation** allows the robots in the swarm to form groups of desired size. If the size of the swarm exceeds the desired group size, it splits into multiple groups.

#### 2.1.4. Miscellaneous

There are further behaviors of swarm robots that fit neither of the categories above.

- ***Self-healing*** allows the swarm to recover from faults caused by deficiencies of individual robots. The goal is thus to minimize the impact of robot failure on the rest of the swarm to increase its reliability, robustness, and performance (see also collective fault detection above). See section 2.2 for more details.
- ***Self-reproduction*** allows a swarm of robots either to create new robots or replicate the pattern created from many individuals. The goal is to increase the autonomy of the swarm by eliminating the need of a human engineer to create new robots. See section 2.2 for more details.
- **Human-swarm interaction** allows humans to control the robots in the swarm or receive information from them. The interaction can happen remotely, e.g., through a computer terminal or proximal in a shared environment, e.g., through visual or acoustic clues.

### 2.2. Detailed Description of Additional Swarm Behavior Categories

In the following, we describe the additional categories of basic swarm behaviors with which we extended the taxonomy by Brambilla et al. (2013), namely: Collective localization, collective perception, synchronization, self-healing, and self-reproduction.

#### 2.2.1. Collective Localization

Collective localization allows the robots in the swarm to find their position and orientation relative to each other via establishment of a local coordinate system throughout the swarm.

##### 2.2.1.1. Sources of inspiration

The approaches given below are engineered without any mentioned inspiration.

##### 2.2.1.2. Approaches

There are two approaches which originate from the multi-robot research domain. First, creating a map of the environment and localizing relative to it. This approach is called simultaneous localization and mapping (SLAM). It can use and merge different sources of information, such as range sensors or visual sensors. Second, using stationary landmarks with known positions and localizing relative to them. To avoid relying on external information, other robots can be used as landmarks. The robots can move alternatingly through the environment while keeping precise localization information. If the initial positions of the robots are known, then also absolute localization is possible. The dead-reckoning approach where robots use odometry for localization is another possibility but introduces an accumulating error which renders it useless for most scenarios.

##### 2.2.1.3. Results

Thrun et al. (2000) present a mapping algorithm where multiple robots can localize in a globally fused map. It requires that the approximate initial positions of the robots are known by all other robots. It uses an incremental expectation maximization approach which allows robots to localize themselves in maps created by other robots. Experiments demonstrate that the robots can localize robustly in real time in large-scale environments using low-end computers. In the follow-up work, the requirement of known initial positions is relaxed, assuming that robots share an overlapping part of their explored maps (Thrun and Liu, 2005). It employs the concept of information filters that represents the robot positions by Gaussian Markov random fields. The robots are able to identify the correct alignments between different local maps by maximizing the correspondence of similar-looking landmark configurations. Fox et al. (2000) present a belief-based approach for collaborative multi-robot localization. It fuses localization information from different sources, such as odometry, environment measurements, and mutual robot detections by combining visual and range sensors. This allows to improve the robots' belief of the world by learning the detection model from data using a maximum likelihood estimator. Experiments demonstrate that a team of robots is superior in localization compared to single robots with a relatively small communication overhead. Kurazume and Hirose (2000) propose the method of cooperative positioning using robots as landmarks. There are two groups of robots that move alternatingly while using the other, stationary group as localization reference. An increased number of robots also increases the redundancy of position information. With the weighted least square method this redundancy decreases the localization error. The authors perform experiments where the robots use range sensors to measure their respective positions. Results show that this localization method performs better than the dead reckoning method including environments with uneven terrain. Rekleitis et al. (2001) propose a method where robots visually observe each other to improve the dead reckoning localization. They propose two algorithms based on triangulation and trapezoidation for small and large-scale environments, respectively. They perform experiments with two robots where one robot carries markers and the other a camera. The results demonstrate that joint localization leads to much more robust localization than odometry alone. When more robots are used the localization precision is increased. Howard et al. (2003) propose a maximum likelihood method combined with a distributed numerical optimization to eliminate the need for landmark robots to be stationary. It combines range measurements with odometry. Robots observe each others motion and exchange this information to create a graph consisting of their positions and respective observations. Experiments with four robots demonstrate that this method is able to localize with adequate precision, and is robust to changes in the environment and to flawed odometry. Furthermore, robots are able to infer the position of other robots they have never seen before. Rubenstein et al. (2014b) apply the robot-landmark approach to a large swarm of 1,024 Kilobots. There are four pre-localized seed robots which define the coordinate system. The other robots localize relative to these seed robots using trilateration of infrared signals. The robots were able to self-assemble and let the swarm morph to a given shape.

#### 2.2.2. Collective Perception

Collective perception combines the data locally sensed by the robots in the swarm into a big picture. It allows the swarm to make collective decisions in an informed way, e.g., to classify objects reliably, allocate an appropriate fraction of robots to a specific task, or to determine the optimal solution to a global problem.

##### 2.2.2.1. Sources of inspiration

Many social insects are able to get a global view using only local information. Examples are honeybees that assess the current global workload balancing by evaluating simple cues like queuing delays (Ratnieks and Anderson, 1999) and ants that use pheromone trails to find shortest paths in large environments (Goss et al., 1989; Hölldobler and Wilson, 1994).

##### 2.2.2.2. Approaches

For collectively determining the type of object observed, the predominant approach is classification of the object among a set of predefined models. Sometimes, the mobility of the robots is used to improve the perception of individual robots. The robots use explicit communication in order to propagate their findings and achieve consensus. The way the robots exchange the information is an important aspect. They have to add contextual information that allows the other robots to correctly interpret the data. Furthermore, the information can be simply forwarded and thus spread in the swarm or modified in order, e.g., to measure the distance to a specific location. There are also approaches from other research domains, such as camera networks (Schranz and Rinner, 2015), but the agents are typically stationary and often centrally controlled.

##### 2.2.2.3. Results

Ye et al. (2002) propose a strategy where sensing agents collect, analyze, and categorize data, enrich it with contextual information, and forward it to synthesizing agents. The latter are then able to use the different aspects observed by the sensing agents to perceive events using an eigen-space method. Using simulation experiments, the authors demonstrate that events can be detected reliably using only the first few eigen values. Kornienko et al. (2005) develop a swarm of micro-robots for a collective classification task. Based on evidence theory, the swarm has to identify the geometries of objects in space by exchanging data from infrared depth sensing while having limited communication capabilities. Experiments show that a wrong belief of an individual quickly converges to the correct belief after exchanging only few messages. King and Breedon (2010) present a simple model of a hexagonal grid world in which a swarm has to differentiate between differently shaped objects. They show that an increased number of agents leads to an overproportional decrease of object detection time. Giusti et al. (2012) present an approach for cooperative gesture recognition with a robot swarm. Each robot processes and classifies camera images locally. Using a distributed consensus protocol, the robots exchange their opinions over a low-bandwidth wireless channel to find a common decision by exploiting the different view points and mobility of the agents. The approach is evaluated through simulation and physical experiments on 13 robots. The results show that the recognition accuracy of the system scales effectively with the number of agents and is robust to communication failures. Stegagno et al. (2014) develop a method that allows a swarm of robots with different types of low resolution sensors to collectively classify objects. Each robot processes the sensor data locally and exchanges its estimation. Using the naive Bayes classifier together with the information received from other robots, the swarm is able to robustly classify objects. The more diverse the sensors are, the better the results. Olfati-Saber and Jalalkamali (2012) present a theoretic framework that employs mobility to improve the information sensed by the swarm using the Kalman-consensus filter. It is employed to track a target with a swarm of agents. Each agent tries to improve its sensing while avoiding collisions with the others. Simulations show that this solution can effectively track linear and non-linear maneuverable targets.

Mazdin and Rinner (2019) present a method for simultaneous coverage of surfaces with a swarm of robots. This method assigns robots to different view points in order to allow effective 3D reconstruction of objects. Simulation results show that this method is able to coordinate the robots while minimizing the mission duration and maximizing the coverage quality. Schmickl et al. (2007) compare different communication strategies for collective perception of a swarm, namely the hop-count strategy and the trophallaxis-inspired strategy. The presented solutions allow a swarm to collectively compare sizes of target areas which are too large for individual robots to perceive. Simulations show that the robots are able to aggregate in the target areas while their numbers are proportional to the size of the target area. Mermoud and Evans (2010) tackle a similar problem in which robots should distinguish good and bad spots using models of chemical reaction networks. They perform experiments with five robots and demonstrate that robots with limited sensing capabilities can collectively achieve good performance.

#### 2.2.3. Synchronization

Synchronization aligns frequency and phase of oscillators of the robots in the swarm. Thereby, the robots have a common understanding of time which allows them to perform actions synchronously.

##### 2.2.3.1. Sources of inspiration

During courtship, males of certain animal species synchronize their behavior. In some firefly species, the phase difference between the blinking of male and female flashing period is important for mating (Buck, 1988). Hence the fireflies synchronize by influencing their flashing phase. Likewise, bushcrickets synchronize or alternate using chirps by altering their chirp periods in response to other chirps (Hartbauer et al., 2005). Igoshin et al. (2001) develop a model that describes the spatio-temporal wave patterns observed from myxobacteria cells. There are many more examples of coupled oscillating systems, e.g., pacemaker cells in the heart (Peskin, 1975) or clapping of spectators in a theater (Néda et al., 2000).

##### 2.2.3.2. Approaches

The oscillators are synchronized to the same frequency with the phases being aligned among the robots in the swarm. Two approaches exist, either the oscillators continuously influence each other to adjust phase and frequency, or they are pulse-coupled meaning that they regularly fire a signal corresponding to their current phase. The latter one is mostly used as it requires fewer interactions between the robots. Robots interact either through acoustic, visual signals or radio communication.

##### 2.2.3.3. Results

Hartbauer and Römer (2006) employ synchronized oscillators as a communication and navigation system. In a synchronized system, robots at a target area increase their frequency and thereby produce phase waves in the swarm that can be used by the robots to perform wave-front navigation, i.e., travel toward higher frequencies. The authors analyze the robustness of the system by simulating up to 300 robots. The results show that this communication system is robust to changes in signal strength, signaling period length, and communication obstacles. Nevertheless, the signaling period is an important parameter to be fit to the scenario. They conclude that pulse-coupled oscillator synchronization is especially suited for swarms of robots as it has low hardware requirements in terms of communication range and processing power. Christensen et al. (2009) apply synchronization to detect faulty robots in the swarm. Using pulse-coupled oscillators and visual signaling, the swarm can determine malfunctioning robots when their phases are not aligned to the rest of the swarm. The authors develop a discrete model that can be applied to robots. Simulations with 100 robots show that robots synchronize faster when they are mobile, synchronization time is linearly proportional to the swarm size where denser networks synchronize faster, and synchronization is robust to communication obstacles but decreases in performance. Experiments with ten physical robots confirm the simulation results, despite the inherent latencies associated with the sensor and actuator systems. In contrast to the bio-inspired approaches, Trianni and Nolfi (2009) synthesize synchronization strategies using artificial evolution. These strategies perform phase coupling between robots in order to allow synchronous movement. Simulations with up to 96 robots show that the strategies scale well and are mainly limited by collision avoidance behaviors. This is confirmed through experiments with up to three robots where sensor and actuator noise is introduced.

Bezzo et al. (2014) synchronize robots in a swarm to determine the network topology and detect changes. They develop a strategy for estimating the degree of oscillator coupling in the swarm and synchronizing them continuously. Applying this strategy to formation control allows three simulated robots to move in a formation. Simulations with five robots show that the network topology can be detected reliably. Barciś et al. (2019) apply the novel concept of swarmalators (O'Keeffe et al., 2017) to robots. This concept couples the oscillator phase with spatial location in such a way that they mutually influence each other. They modify the original model taking into account the discrete nature of robots. Simulations of 100 robots and experiments with 10 robots show that the spatio-temporal patterns can be performed in stationary and dynamic scenarios. Perez-Diaz et al. (2015) perform a case study to analyze how motion and sensing capabilities influence the synchronization capabilities of a robot swarm. By altering the field of interaction (e.g., camera field of view) and the speed at which the robots travel, the emergence of synchrony can be influenced. The robot speed influences the time until synchrony is reached whereas a narrow field of interaction results in a low degree of synchronization. Furthermore, high robot densities limit the synchronization possibility due to signal occlusion and robot collisions.

#### 2.2.4. Self-Healing

Self-healing allows the swarm to recover from faults caused by deficiencies of individual robots. The goal is to minimize the impact of robot failure on the rest of the swarm to increase its reliability, robustness, and performance. After detecting the fault, appropriate countermeasures must be taken.

##### 2.2.4.1. Sources of inspiration

The immune system of vertebrates shows how biological systems protect complex organisms against diseases. This serves as inspiration for the artificial immune system (AIS). Timmis et al. (2010) give an overview of how AISs and swarm intelligence relate. They point out many similarities and conclude that both systems are complementary tools for solving complex engineering problems. Regeneration in biological systems allows animals to self-heal their body, e.g., salamanders, starfish, and lizards are able to regenerate lost limbs (Wallace, 1981). Another prominent example is the morphallaxis, i.e., tissue regeneration of Cnidarian hydra Shimizu et al. (1993). It exhibits what is sometimes referred to as scalable self-healing: When the hydra is dissected, each part can self-heal into a fully functional and independent hydra where its size is proportional to the number of cells.

##### 2.2.4.2. Approaches

There are two ways to tackle the problem of self-healing. First, healthy robots can aid the faulty robots in recovering. It requires an explicit failure management routine which is typically inspired by the immune system. Second, the swarm can adjust its behavior while ignoring failing robots. This does not require any special handling of the failure case. It is typically inspired by biological regeneration.

##### 2.2.4.3. Results

As self-healing is a relatively challenging topic for swarm robotics, only few embodied studies exists and most work is done through simulation experiments. Dai et al. (2006) present a model for detecting and healing software components of swarm robots. It is part of the NASA autonomous nano technology swarm (ANTS) concept mission (Vassev et al., 2012). This model is only partly distributed as it relies on a central cyber disease library. Each robot runs one or more virtual neurons as background processes. They monitor certain system variables, such as CPU, memory, or network usage. In case of anomalies, it freezes the process in question and reports its behavior to a higher-level controller. It can perform further diagnosis, e.g., by assigning more neurons, and generate a prescription based on the cyber disease library. The prescription is applied by the executor process which reports back results. This allows the cyber disease library to learn and improve prescriptions. In case the prescription does not work, further escalation steps are possible, such as killing the faulty process or even rebooting the whole machine. A simulation case study shows that a memory leaking process is successfully detected and eventually killed. The results show that the system becomes more reliable and robust against failures and failure propagation. Even though faulty processes degrade the overall system performance, the performance improves compared to systems without the self-healing properties. Timmis et al. (2016) apply self-healing to overcome hardware failures. They present a solution that is inspired by granuloma formation, a process of containment and repair found in the immune system. They apply it to a swarm of robots performing flocking and taxis toward a beacon. When a robot has a discharged battery and loss of mobility, it would anchor the whole swarm which would then fail to reach the beacon. The proposed solution allows energy sharing between healthy and faulty robots. The faulty robots signal their need of help to the other robots within range. Depending on the required energy and the energy available at the healthy robots, a varying number of robots surround the discharged robots to share energy. Other robots ignore this robot cluster and regard it simply as an obstacle, continuing their mission. Simulation experiments with 10 robots show that the granuloma formation algorithm works well even when half of the robots in the swarm are experiencing low energy levels. Other algorithms are compared where only the nearest healthy robots perform the healing. They fail to heal the swarm when three or more robots have a discharged battery.

A broad body of research is directed toward pattern formation and morphogenesis in self-healing. Cheng et al. (2005) propose the SHAPEBUGS approach where agents evenly disperse within a predefined shape. First, the agents perform trilateration to establish a common coordinate system. This is aided by allowing a few agents to know their initial position. Then the agents use the contained gas model to move in a way that they are equally spaced within the desired shape. In case of agent failure, the other agents simply adapt their positions to again reach an equilibrium density. The agent model contains a proximity sensor and wireless communication to exchange positions. It furthermore requires a compass to determine the global orientation of the agents. Simulations show that the swarm can self-repair and restabilize in cases of agent death or displacement. Furthermore, it can overcome large degrees of sensor and movement errors of the agents. Rubenstein and Shen (2008) relax some of these assumptions and still achieve similar results. The model differs in that the robots build compact shapes. Thereby, the size of the shape varies rather than its density. The model requires only a single sensor for local information and communication. The shape is given to the robots as a potential field where the robots aim to move to its center while avoiding collisions. The scale of the shape is calculated as function of the estimated swarm size. Additionally, each robot changes its color to a color that is pre-defined depending on the position. Thereby, the robots can form colored patters or displays. When properly synchronized, they can even show time varying patterns. Simulation results show that the robots can perform scalable self-healing by recreating the desired shape for varying swarm sizes. In later work, Rubenstein et al. (2014b) demonstrate this on a large swarm of physical robots as described above. Arbuckle and Requicha (2010) propose a model where the robot swarm builds shapes by arranging on the boundaries of a polygon. They propose an external compilation process that uses the polygon to derive a set of parameterized local rules to be executed by a swarm of homogeneous, stateless robots. By attaching physically to each other, the robots can communicate directionally. The agents stay connected as long as they are communicating. By replying with predefined messages, the state of the system is “externalized” in the circulating messages. By attaching to each other, the agents grow the edges of the polygon while randomly wandering robots “diffuse” into the interior of the polygon by replacing boundary robots that in turn move into the polygon interior. Simulation experiments show that the swarm can build simple polygons. It can heal from failures due to communication faults, such as dropping messages. Robots that do not communicate anymore, drop out of the shape and are replaced by new ones. If the shape is broken into two, the swarm creates two shapes with the original size.

#### 2.2.5. Self-Reproduction

Self-reproduction allows a swarm of robots either to create new robots or replicate the pattern created from many individuals. In the first case, the robots produce identical copies of themselves. The goal is to increase the autonomy of the swarm by eliminating the need of a human engineer to create new robots. In the second case, the robots copy a structure consisting of many individual robots. Existing approaches are not fully autonomous yet and typically require at least the building blocks to be provided to the swarm. In contrast to self-reconfiguration of formed patterns, the goal of self-replication is to assemble a functional robot from passive components.

##### 2.2.5.1. Sources of inspiration

All biological organisms possess the ability to reproduce, either sexually or asexually.

##### 2.2.5.2. Approaches

The theory of self reproducing machines already exists for several decades, e.g., von Neumann (1966) introduced the idea of an automaton model for self-reproduction. The research in this direction followed the general idea of template-replicating systems, i.e., to create a new robot according to an existing model. Gross and Dorigo (2008) give an historic overview of the development in this direction. Other approaches follow the evolutionary design strategy. The existing approaches assume the robot hardware to be modular in order to have base building blocks (Yim et al., 2002). The finer the modules, the more difficult the process, but the more flexibly a new robot can be created.

##### 2.2.5.3. Results

Lipson and Pollack (2000) evolve the design for simple electromechanical systems through simulation experiments. The building blocks are bars and actuators that are connected through joints and controlled by artificial neurons. The performance of the evolved systems is measured in terms of the distance it is able to locomote. The best performing designs are fabricated using rapid, additive manufacturing technology. This process allows robots to design new robots with minimized human intervention. The manufactured robots are able to locomote similarly to the simulation models. Suthakorn et al. (2003) present a fully autonomous, self-replicating robot built from Lego parts. It assembles a new robot from four pre-assembled subsystems that hold together using magnets and shape-constraining blocks. The controller of the replica is already pre-programmed with the same program as the original. Experiments show that the original robot, which is guided by lines on the ground, is able to detect the subsystems and assemble them into a fully functional replica. Zykov et al. (2007) present the design of a modular robot cube, called Molecube. These cubes can attach to each other using electromagnets and hence form complex patterns. These cubes have one actuated degree of freedom to control the shape of the assembled pattern. The authors present experiments where the spatial patterns and corresponding controllers are both manually created and automatically designed with artificial evolution. The results show that the swarm of Molecubes reproduces identical copies of its pattern, both in simulation and physical experiments. The only human interaction is by providing enough Molecubes as building material. The authors conclude that the more units are involved and the simpler and more homogeneous they are, the more information is being reproduced by the system itself, as compared with information pre-existing in the parts and environment.

## 3. Swarm Applications

Even though swarm robotics is a relatively young field of research and has not been widely accepted in industry, this section is a first collection of existing applications and attempts at swarm robotic products. Swarm robotic researchers have designed and developed a number of platforms to test and analyze swarm algorithms. In their publications the authors always stated their attempt to envision future industry applications (Sharkey, 2007) out of the simplicity of swarm robotic research platforms. Thus, we split our survey into swarm robotics research platforms (Table 1) and industrial projects and products (Table 2). The industrial projects and products are mainly listed to serve as application examples in real-world environments above a technology readiness level (TRL) of four (Héder, 2017) where the validation of the platform is already in the relevant environment. The research platforms enable researchers to verify, demonstrate, and experiment with swarm algorithms in laboratory environments, thus on a TRL of maximum four.

**Table 1:**
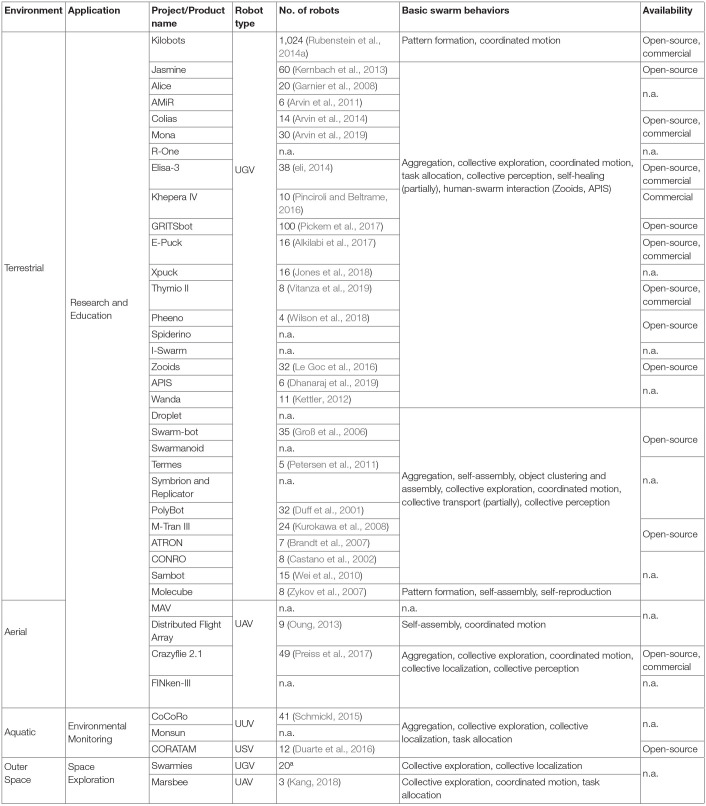
Classification of research platforms for swarm robotics.

*^a^Project's official website: http://nasaswarmathon.com/*.

**Table 2:**
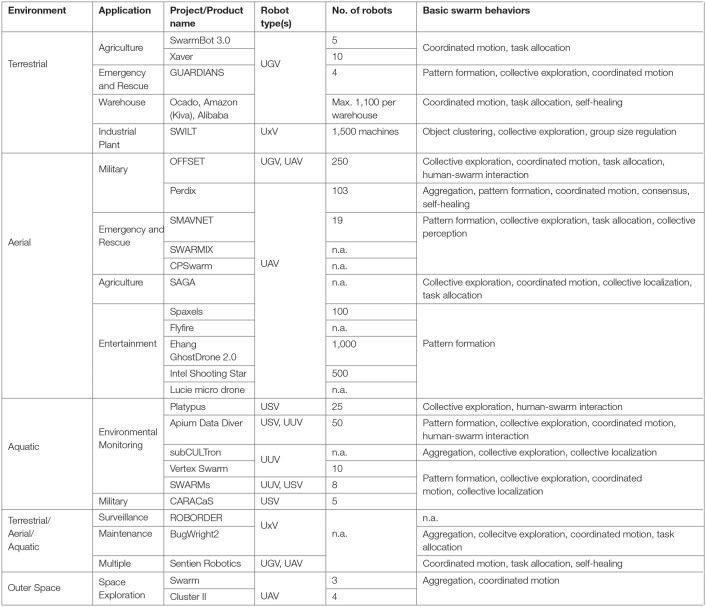
Classification of industrial projects and products.

Both, research platforms and industrial projects and products are categorized according to the environment they are used in: terrestrial, aerial, aquatic, and/or space. All tables list the type of the application, the project or product name, the type of robot, the number of robots in the swarm, and the basic swarm behaviors corresponding to the definitions of section 2. The type of robot corresponds to one of the following categories: unmanned ground vehicle (UGV), unmanned aerial vehicle (UAV), unmanned surface vehicle (USV), unmanned underwater vehicle (UUV), or in general as UxV. The number of research platforms used in a swarm is proven with specific research publication. For the industrial swarm applications we refer the reader to the project's or product's website for this information. For each research platform, industrial project and product we list one or more basic swarm behaviors because no project or product uses integral swarm algorithms. They rather use parts of the algorithms, and adapt them to the underlying application. Additionally, the table for the research platforms differentiates between open-source (in hardware and software) and/or commercially available. We do not classify the swarm robotic platforms and products related to their price, dimension, and number of usage in different research/engineering projects.

The focus of this collection is on basic swarm behaviors embodied on robots. Therefore, we only list projects or products that are based on a robotic platform. We neglect research projects that focus solely on swarms with a purely theoretical or virtual nature.

### 3.1. Research Platforms

This section presents research platforms that are developed for educational and scientific purposes, summarized in Table 1. They allow to investigate the application of swarm algorithms to robots. Note, that other sophisticated robotic research platforms exist which are not included, as they are not developed with the intention of using them in swarm applications, e.g., the Balboa robot kit^1^.

#### 3.1.1. Terrestrial

The Kilobots swarm (Rubenstein et al., 2014a) is probably the best known swarm of robots developed for research and education. Kilobots are very small with a diameter of 33 mm, the locomotion is based on vibration motors and the communication is implemented using infrared light reflecting off the ground. They became very famous for their self-assembling capability forming different shapes with a swarm of 1,024 Kilobots (Wyss Institute, 2017). The Kilobot is available open-source^2^ or commercially at K-Team^3^.

Jasmine is another widely used swarm robotic platform. The open-source^4^ platform was mainly built for large-scale swarm robotic experiments equipped with a series of sensors, including touch, proximity, distance, and color sensors. The intention for large-scale swarms was also pursued with the swarm robotic platform Alice (Caprari et al., 1998). Many additional sensors including a linear camera can extend the basic capabilities. A series of research platforms building upon each other is given with AMiR (Arvin et al., 2009), Colias (Arvin et al., 2014) (open-source^5^ and commercially^6^ available), and Mona (Arvin et al., 2017) (open-source^7^ and commercially^8^ available). The platform R-One (McLurkin et al., 2013) is also designed for the usage as swarm robotic platform. Although it uses a camera tracking system for ground-truth localization, and server software to connect all the pieces together, several experiments “close” to swarm intelligence can be performed. The Elisa-3 swarm robotic platform, open-source and commercially^9^ available, also uses an Arduino microcontroller with a high number of sensors including eight IR proximity sensors, three-axis accelerometer, and four ground sensors. The robot is able to recharge autonomously using a charging station. The robots in the swarm communicate using either IR or radio. The Khepera IV (Soares et al., 2016) is designed for any indoor lab application. A Linux core, color camera, WLAN, Bluetooth, USB Host, accelerometer, gyroscope, microphone, loudspeaker, three top RGB LEDs, and improved odometry makes it a compact and complete research platform for swarms in different scenarios. The Khepera IV is commercially available at K-Team^10^. The GRITSbot (Pickem et al., 2015) is the open-source^11^ swarm robotic platform used in the Robotarium^12^ at Georgia Tech, Atlanta. The Robotarium provides remote access to a large team of robots. Scholars can upload code to run experiments remotely to collect data. Features like automatic registration of robots with a server, autonomous charging, wireless code upload to the robots, and automatic sensor calibration makes the Robotarium attractive for remote research experiments. All these platforms use wheels for their locomotion and a set of different sensors, including distance and light sensors.

The e-puck robot (Mondada et al., 2009), and its successor e-puck2, are designed as educational and research robots to make it easy to program and control the robots' behaviors. It uses diverse sensors, e.g., infrared proximity sensors, a CMOS camera, and a microphone. The e-puck is available open-source^13^ or commercially at GCtronic^14^. The Xpuck is an extension of the e-puck in terms of aggregate raw processing power (as used in modern mobile system-on-chip devices) of two teraflops. Thus, higher-individual robot computation can be achieved, e.g., image processing using the ArUco Marker tracking (Jones et al., 2018). The Thymio II robot (Riedo et al., 2013) targets the understanding of programming and robotic concepts using a wide range of sensors, including temperature, infrared distance, accelerometer, and microphone. Programming can be done in Blocky using visual or text-based programming. The Thymio II is available both open-source and commercially at Thymio^15^. A recent platform for open-source^16^ swarm robotics education and research purposes is called Pheeno (Wilson et al., 2016). The user can adapt the platform with custom modules in three degrees of freedom. To interact with the environment it uses IR sensors. The Spiderino platform (Jdeed et al., 2017) is a six-legged open-source^17^ robot with spider-like locomotion. It is based on a hexpod toy that is enhanced by a PCB including an Arduino microcontroller, a WLAN module, and several reflective infrared sensors.

The goal of the I-Swarm (Intelligent Small-World Autonomous Robots for Micro-manipulation) project is the development of micro robots to form a swarm. The robot has a small size of only 3 × 3 × 3 mm^3^, is solar powered without battery, performs locomotion via vibration, and communication via infrared transceivers (Seyfried et al., 2004). It has been developed with the goal to build a swarm of 1,000 robots (Seyfried et al., 2004). Prototypes of this robot can be seen in the technical museum in Munich, Germany.

The idea behind the open-source swarm robotics platform Zooids^18^ is different: It handles both the interaction and the display, and thus offers a new class of human-computer interfaces. The swarm is controlled via light patterns projected using an overhead projector (Le Goc et al., 2016). The APIS^19^ (Adaptable Platform for Interactive Swarm) comprises several components: the swarm robotic platforms, the infrastructure and test environment for the swarm, and the software infrastructure and simulation (Dhanaraj et al., 2019). The focus are experiments related to human-swarm interaction. For these interactions, additionally to sensors, the platform is equipped with an OLED display and a buzzer. The Wanda (Kettler et al., 2012) swarm robotic platform has a special assembly that could be used, e.g., to clean up the environment with a swarm. In addition, the authors implemented a whole tool chain especially for these robots from design and simulation to deployment.

The Droplet (Klingner et al., 2014) is another swarm robotic platform for teaching and research. It is a spherical robot which is able to organize into complex shapes with its neighbors by using vibration locomotion. It charges and communicates via a powered floor that is equipped with alternating stripes of positive charge and ground. It is available as an open-source project^20^. The Swarm-bots (Mondada et al., 2002; Groß et al., 2006) can configure themselves to different geometric 3D shapes. The robots are constructed by a number of simpler, insect-like robots, which are built of relatively cheap components (the design is open-source^21^). A swarm of these robots is capable of self-assembling and self-organizing to adapt to its environment. With this assembling capability the swarm is able to transport objects that would be too heavy for the individual robots. The successor of the Swarm-bots project is the Swarmanoid project, which represented the very first attempt to study the integrated design, development, and control of a heterogeneous open-source^22^ swarm robotics system. The swarm in the Swarmanoid project covers autonomous robots of three types (each with an additional set of sensors): eye-bots (UAVs that can attach to an indoor ceiling), hand-bots (UGVs capable of climbing), and foot-bots (UGVs capable of self-assembling) (Dorigo et al., 2013). The Termes robots (Petersen et al., 2011) collaborate without communication or GPS localization to create large structures using modular blocks. The underlying concept is stigmergy and is inspired by the way termites build their nests in nature. The Termes robots are block-carrying climbing robots that can create these structures in unstructured environments. Symbrion and Replicator (Kernbach et al., 2008) are two sister projects, that develop autonomous platforms for usage in swarms. They can be operated either individually or form special patterns by physically connecting to each other. The main goal of these projects is to create a road-map of how to achieve the evolvability of robot organisms. PolyBots (Duff et al., 2001) are self-reconfigurable robots. Various types of locomotion capabilities and object manipulation modules are interchangeable that allow to form a number of shapes, e.g., an earthworm type to slither through obstacles, or a spider to stride over hilly terrain. These robots find their application if the environment is unknown or if robots need to perform multiple tasks. Further modular robots that allow self-configuration with similar robotic technologies include M-TRAN (Murata et al., 2002), M-TRAN II (Kurokawa et al., 2003) and M-TRAN III (Kurokawa et al., 2008) (available as open-source^23^ project), ATRON (Brandt et al., 2007) (available as open-source^24^), CONRO (Castano et al., 2002), Sambot (Wei et al., 2010), Molecube (Zykov et al., 2007), but to name a few^25^.

#### 3.1.2. Aerial

There are several miniature and micro UAVs which form a good basis for a swarm system of inexpensive robots for research and education. An overview of such small-scale UAVs can be found in the publications by Cai et al. (2014) and Swetha et al. (2018). Although multiple off-the-shelf Micro Air Vehicles (MAVs) exist and are quite popular in the games industry, and in businesses for video- and photography, they typically have closed flight controllers that do not allow to develop custom algorithms (e.g., Qualcomm Flight Pro^26^, DJI M100^27^). One UAV designed specifically for usage in swarms is the MAV presented by Roberts et al. (2007). The MAVs are equipped with three rate gyroscopes, three accelerometers, one ultrasonic sensor, and four infrared sensors. It has been developed within the Swarmanoid project (Dorigo et al., 2013). A very distinct research platform is the Distributed Flight Array (Oung and D'Andrea, 2011). Each UAV makes up a module of a larger array and has a single rotor only. The modules self-assemble into a multi-rotor system where all vehicles must cooperate for coordinated flight. To facilitate this, they exchange information among each other and adjust local parameters. With the Crazyflies, available both open-source and commercially at bitcraze^28^, a swarm of UAVs can be realized indoors. They use multiple sensors, e.g., accelerometer, gyroscope, magnetometer, and a high precision pressure sensor (Preiss et al., 2017). Their low weight of 27 g allows experiments with reduced danger for humans. The Crazyflie's localization relies on external tracking systems, such as OptiTrack^29^. Another indoor swarm can be build with the FINken-III (Heckert, 2016)^30^ and its predecessors. They use optical flow, infrared distance, and a tower of four sonar ranging sensors.

#### 3.1.3. Aquatic

In the CoCoRo (Collective Cognitive Robotics) project (Schmickl et al., 2011) a swarm of 41 heterogeneous UUVs has been developed. There are three types of vehicles: A base station USV, an exploration UUV, and UUV for relaying information between the explorers and the base station. Communication is performed with sonar and electric fields. The main applications envisioned are environmental monitoring, measuring water pollution and effects of global warming. The UUV Monsun (Osterloh et al., 2012) uses two types of communication: an acoustic underwater modem for information exchange and a camera to recognize and follow other swarm members. CORATAM (Control of Aquatic Drones for Maritime Tasks) (Christensen et al., 2015) is a project that develops swarms of USVs. Envisioned applications are environmental monitoring, sea life localization, and sea border patrolling. The platforms are available open-source^31^ and can execute swarm algorithms generated using evolutionary computation (Duarte et al., 2016).

#### 3.1.4. Outer Space

For space exploration, NASA has developed Swarmies^32^ to collect material samples, such as water, ice, or useful minerals on Mars. This application is referred to as *in-situ* resource utilization (ISRU). Simultaneously, NASA launched a swarmathon^33^ to entice students to develop swarm algorithms based on ant foraging. In an experiment 20 Swarmies could travel 42 km of linear distance in 8 h. The same distance was covered by Mars rover Opportunity in 11 years. Another innovative project was accepted by the NASA Innovative Advanced Concepts (NIAC) program. The objective is to increase Mars exploration using a swarm of Marsbees (Kang, 2018). These are robotic flapping wing flyer the size of a bumblebee. They are to self-explore the environment and use the Mars rover Opportunity as base and charging station. During the NIAC funding, a concept for the technical implementation of the flapping flyer using insect-like wings will be proposed.

### 3.2. Industrial Projects and Products

#### 3.2.1. Terrestrial

One of the biggest challenges in agriculture is the increasing demand for food production (Tilman et al., 2011). SwarmFarm Robotics^34^ is a company that provides farmers with swarms of agricultural UGVs—the SwarmBot3.0. They work cooperatively, but follow a centrally planned schedule. Before starting the mission, a given field is decomposed into smaller cells which are then allocated to the vehicles (Ball et al., 2015). The swarm's tasks are diverse, but involve planting, applying fertilizer, eliminating weeds and insects, irrigation, and harvesting. A similar project is addressed by the Fendt company with the UGV Xaver^35^. Each Xaver is a battery-operated planting UGV that operates cloud-controlled and collaborates with the other UGVs in the swarm in terms of a centrally planned seeding plan (Blender et al., 2016). The series production of the robots started with the EU-project MARS (Mobile Agricultural Robot Swarms).

Within the GUARDIANS (Group of Unmanned Assistant Robots Deployed in Aggregative Navigation by Scent) project (Saez-Pons et al., 2010), a swarm of autonomous UGVs has been developed for emergency and rescue applications. This swarm can be used in dangerous situations where toxins are released that severely impair human senses. The robots warn of toxic chemicals, provide and maintain mobile communication links, infer localization information, and assist in searching. They can generate a formation and navigate while keeping this formation using so-called social potential fields. All tasks can be achieved without central control, and some of the behaviors can be performed without explicit communication between the robots.

Ocado (Telegraph, 2018) is an automated warehouse that uses a swarm of homogeneous cuboid UGVs. Grocery orders are assembled and dispatched using 1,100 collaborative robots. The robots collect ordered crates of food from stacks beneath a huge metal grid organized like a chess grid and deliver them to chutes, where human workers put the customer orders together. As the crates are organized in piles, the robots assist each other to lift out the ones standing in the way. They are operated in bursts to ensure breaks for charging. The robots are controlled centrally by a cloud server. Data is transmitted between the robots and the cloud using cellular technology. The cloud server handles the vast amount of data to coordinate the robots using machine learning approaches. The biggest player for robot swarms in warehouses is Amazon using the Kiva robot system (Brown, 2018). It uses up to 100,000 robots worldwide to move shelf towers in its warehouses. The robots use motion sensors to recognize other robots or shelves in their way. To find their way to the human workers who assemble the customer orders, they use visual tags on the ground of the warehouse to localize and navigate using the A^*^ algorithm. The dispatching is organized centrally and communicated to the robots using WLAN. Robots drive to a charging station automatically in case of low power. A very similar system is used, among others, by the retailer Alibaba (Pickering, 2017).

The SWILT (Swarm Intelligence Layer to Control Autonomous Agents) project (Khatmi et al., 2019)^36^ takes another approach to modeling UxV. The project focuses on industrial plants in the semi-conductor industry with a high-product mix (about 1,500 different products), where the swarm is made up of lots, machines, and other equipment. The innovation in SWILT is to apply nature-inspired behaviors extracted from swarm intelligence algorithms to the individuals of the swarm instead of pre-calculating global schedules or routing tables. The main difference to traditional methods, such as linear optimization is that feasible, global solutions emerge from local behavior.

#### 3.2.2. Aerial

Swarm missions in the air are typically covered by UAVs, and can be used for different applications. For example, military applications are represented by the OFFSET (OFFensive Swarm-Enabled Tactics) project (Chung, 2017). The main idea of this project is to enhance reconnaissance with UAVs and UGVs inside cities. The applied robots should identify threats using more than 100 different swarm tactics in a game-based environment. The United States Air Force (USAF) works on a swarm of 250 UAVs that is able to perform a 6-h mission for the reconnaissance of eight city blocks. Another swarm of UAVs in military applications is funded by the Pentagon: the Perdix drone (Mizokami, 2017). A swarm of 103 Perdix UAVs is released from three F/A-18 Super Hornets. The swarm of Perdix drones is able to perform four different missions, including hovering over a target, or forming a 100-m-wide circle in the sky. Their swarms have no central control, no leader, adapt to UAVs entering or exiting the team, are not pre-programmed, make collective-decisions, and can fly in formation. Typical military applications include surveillance missions and targeted assassinations.

The SMAVNET (Swarming Micro Air Vehicle Network) project (Hauert et al., 2009) belongs to the application domain of emergency and rescue. A swarm of autonomous MAVs is developed to deploy and manage an *ad-hoc* WLAN network (Varga et al., 2015). The application is to connect and coordinate rescue teams. Another aim is the exploration of disaster sites, with the goal of localizing victims and directing rescuers toward them. In the project SWARMIX^37^ they form a swarm of heterogeneous agents (humans, dogs, UAVs) that work cooperatively in a search and rescue mission (Flushing et al., 2014). Similar goals related to search and rescue were achieved in the CPSwarm project^38^, although the focus was on developing a toolchain for CPS swarm design, modeling, simulation, and deployment.

For agriculture, the SAGA (Swarm Robotics for Agricultural Applications) project^39^ targets a distributed monitoring and mapping scenario using a swarm of UAVs, which is a novelty in smart farming (Albani et al., 2019). The fitness of the swarm is measured as a trade-off between exploration and weed recognition time. An on-board vision system is used to detect weeds.

Nowadays, entertainment in terms of light shows is a very attractive application for swarms of UAVs. The UAVs are equipped with LED lights and perform different pattern formations creating a free-form display light show, typically accompanied by music. Most providers, like Spaxels^40^, Flyfire^41^, Ehang^42^, Intel (Barrett, 2018), and Lucie micro drone^43^ have central solutions: the “swarm” of up to around 1,000 UAVs is controlled centrally and follows pre-planned patterns.

#### 3.2.3. Aquatic

Environmental monitoring is a common application for swarms in aquatic missions. Platypus (Jeradi et al., 2015) sells autonomous swarm robotic boats, so-called USVs, to measure and monitor water quality. They provide dense maps of defined bodies of water to give a comprehensive picture of the water quality including salinity and oxygen stratification. Different platforms are used depending on the scale and type of the body of water. The boats perform centrally planned collective exploration and interact with the human operator using team oriented planning (Farinelli et al., 2017). Another example is the Apium Data Diver^44^. It is a prototype vehicle built for operations in swarms on surface and under water. It is able to dive to a maximum depth of 100 m and has multiple sensors on board including temperature, pressure, and GPS. Possible applications include oceanography, aquaculture, hydrographic survey, and defense. The Data Diver swarm can receive high level commands from a human operator for navigating to a target and forming specific patterns (MacCready, 2015). Further autonomous UUVs were developed by Hydromea^45^. Their swarm of UUVs, the so-called Vertex swarm, is able to take water quality measurements at many locations simultaneously down to a depth of 300 m and create 3D data sets with high spatial and temporal resolution much faster than traditional methods. The small size of the UUVs allows for their application, e.g., under ice, in protected areas, underground water caverns, and storage tanks. The UUVs are able to localize in the swarm using acoustic triangulation. Using this positioning information, they form an underwater *ad-hoc* communication network (Schill et al., 2016). The main goal of the SWARMs (Smart Networking Underwater Robots in Cooperation Meshes) project (Real-Arce et al., 2016)^46^ is to make underwater and surface vehicles more accessible and useful for maritime and offshore operations. The aim is to extensively use maritime vehicles instead of professional divers for the typically dangerous offshore operations. SWARMs mainly works on the design and development of a set of software and hardware components to incorporate them into the current generation of maritime vehicles. This helps to improve autonomy, cooperation, robustness, cost-effectiveness, and reliability. Exemplary applications of SWARMs comprise among others: corrosion prevention in offshore installations, monitoring of chemical pollution, and tracking of plumes. A major research focus lies on reliable underwater communication (Rodríguez-Molina et al., 2017) leveraging topology control (Li et al., 2017).

Another application in aquatic environments is military. The software kit CARACaS (Control Architecture for Robotic Agent Command and Sensing), initially developed by NASA for the Mars rover, has been adapted by the Office of Naval Research (ONR). This technology allows autonomous operation of US Navy boats where these USVs interact with each other (Smalley, 2016). These characteristics allow the swarm of USVs to choose their own routes, to intercept enemy vessels as a swarm, and to escort and protect naval assets. To support changes in the swarm, CARACaS allows to re-plan and distribute new task lists. The first successful demo in 2014 has been held on the James River in Virginia, where CARACaS was installed on multiple rigid-hulled inflatable boats. The main application has been demonstrated during the Safe Harbor mission (Hsu, 2016). The project subCULTron (Submarine Cultures Perform Long-Term Robotic Exploration of Unconventional Environmental Niches)^47^ uses the results of the CoCoRo project (described in the previous section Research Platforms) to deploy and test a swarm of UUVs in the Venetian Lagoon in Italy to evaluate the learning, self-regulation, and self-sustainability of the swarm.

#### 3.2.4. Terrestrial/Aerial/Aquatic

The project ROBORDER (Autonomous Swarm of Heterogeneous Robots for Border Surveillance)^48^ employs an autonomous swarm of heterogeneous robots (UGV, UAV, USV) equipped with multimodal sensors for sea and land border surveillance. Their aim is to detect and identify criminal activities in a vast heterogeneity of threats. The main objective of this project is to incorporate multimodal, statically networked sensors in a swarm of robots.

The project BugWright2^49^ focuses on service and maintenance of large ships. This includes hull cleaning and inspection. Typically, this induces high costs. Therefore, the project's objective lies in the deployment of different cooperating UxV swarms (UGV, UAV, UUV) for a detailed multi-robot visual and acoustic inspection of the hull structure, detecting corrosion patches, and cleaning the surface where necessary.

Sentien Robotics^50^ develops UGVs and UAVs to serve the needs of surveillance, environmental monitoring, infrastructure inspection, and national security. The company develops scalable swarm intelligence software, sensor data processing algorithms, and robot hardware. It furthermore develops a system for automatic launching and recovering of multiple UAVs (Borko, 2016).

#### 3.2.5. Outer Space

Two swarms of satellites are currently in Earth orbit for space exploration: Swarm (Agency, 2004) and Cluster II (Escoubet et al., 2001). Swarm has been launched in 2013 and consists of three identical satellites, each 9 m long, and placed into two different polar orbits: two side by side at an altitude of 450 km and a third at an altitude of 530 km. Their primary task is to study the Earth's magnetic field. Cluster II has been launched in 2000 and consists of four identical, cylindrical (2.9 × 1.3 m) spacecraft flying in a tetrahedral formation. Their task is to study the impact of the Sun's activity on the Earth's space environment. This is the first time a mission is able to provide three-dimensional data on the influence of the solar wind on the Earth's magnetosphere.

### 3.3. Discussion

In the above sections we provided an overview of currently available industrial projects and products in the area of swarm robotics as well as research platforms for swarm robotics. This provides researchers and engineers in the domain of swarm robotics a comprehensive overview of current work, existing products, ongoing projects, and available research platforms. From this overview it can be seen that swarm robotic applications are still rare nowadays. Often, the swarm size depends on the number of robots that companies or research agencies have in stock, and are not always selected according to the desired swarm behavior. Although research has been going on for several decades, a breakthrough of swarm robotics, especially for industrial applications, has not yet occurred.

This is because there are still several open issues. First of all, the dependability of the robot swarm is a concern. Natural swarms work with the assumption that individual swarm members might fail. In engineered swarms, high reliability and availability is desired in order to provide a working system. Failure of individual swarm members can increase the operational cost and can lead to safety issues, especially for UAVs. Swarm behaviors with their emergent characteristics executed by autonomous robots relying on distributed information cannot give the required guarantees on safety, security, and availability. Therefore, many industrial projects still rely on centralized control, e.g., in the agriculture and warehouse applications of section 3.2.1, or the entertainment applications in section 3.2.2. In these projects, the term swarm is solely used to imply the high number of agents. The implementations neglect the principal idea of swarm robotics which is distributed decision making that leads to self-organized behavior. Even though the robots have the ability to identify their environment, gather data locally, and communicate this data with the rest of the swarm, they rely on a central unit. This central unit either pre-defines the behavior of each individual robot or, in more dynamic scenarios, processes the information received from the robots to control their behavior. The issue of safety and security is addressed in research projects like SAGA (Albani et al., 2019), SWILT (Khatmi et al., 2019), and CPSwarm (Bagnato et al., 2017). In these projects additional routines are defined that allow to stop individual swarm members or the entire self-organized swarm to prevent harm to humans or other machines. This is achieved by running multiple processes in parallel to react to certain events. For example, sensor data or emergency stop signals by a human operator can be processed in parallel to the behavior algorithm in order to immediately stop any movement of the swarm members. These emergency processes provide a deterministic behavior as opposed to the normal run time behavior. The safety issues are less critical in aquatic environments. The USVs and UUVs described in section 3.2.3 are able to perform fully autonomous exploration and localization. Compared to terrestrial or aerial environments, the possibility of harming humans is relatively low. Though, harm to the environment or animals is not taken into account.

Another issue is the communication within the swarm and between the swarm and a central unit commanding and controlling station. For a swarm to work fully autonomously, it should provide its own means of communication. This is achieved by *ad-hoc* WLAN networks typically employed in emergency and rescue scenarios (see section 3.2.2). Such communication networks have a limited range and are less stable since they can break down when individual robots fail or move out of range. An infrastructure-based network, such as a cellular network, can provide more stable communication but it requires the installation of base stations. While this is typically available for terrestrial or areal environments, this does not work for space or aquatic missions. Especially in aquatic missions, commonly used radio communication does not work due to the high attenuation of water (Rodríguez-Molina et al., 2017). Therefore, robot swarms in such environments must use less researched technologies, such as sound or electric field communication that have lower throughput (see section 3.1.3). Besides the technological limitations, an important issue when communicating is security. This is of special interest in military applications (section 3.2.2). First, information exchanged between robots could be sensitive and should not be disclosed to hostile parties. Second, the behavior of a swarm could be influenced based on the information the swarm members receive. This means that the behavior of a swarm could be influenced by altering the messages exchanged between robots or injecting false information in the swarm. Therefore, the communication in the swarm needs to be encrypted and swarm members must authenticate against each other in order to provide reliable behavior. This is especially important when a central station sends commands to control the swarm.

Compared to the industrial projects and products, the swarm robotic research shows swarm behaviors close to the natural swarm inspiration that relies on distributed control. Generally, the research platforms' hardware design technologically follows the inspiration from nature as it is small and cheap. They typically use simple and reduced microcontrollers, such as the Arduino. In terms of other hardware characteristics, they are quite diverse. Especially the UGVs show different types of locomotion, e.g., the Kilobots (Rubenstein et al., 2014a) use vibration, the Spiderino (Jdeed et al., 2017) is a six-legged robot, and the AMiR (Arvin et al., 2009) and its successors use wheels. Furthermore, they show different types of communication, e.g., the Kilobots (Rubenstein et al., 2014a) use reflecting light, the I-Swarm (Seyfried et al., 2004) uses infrared, and the Khepera IV (Soares et al., 2016) uses WLAN. Additionally, different types of power sources are used, e.g., batteries (which are most common) and solar cells for the I-Swarm (Seyfried et al., 2004). Besides the diverse actuators, they offer a number of different sensors that can be used for different swarm behaviors to interact with the environment. As outlined by the authors, these platforms are mainly used for research. So, most circuit designs are published open-source online. Partially, these platforms are specifically dedicated for educational use. For example, the Thymio (Riedo et al., 2013) is offered online with a lot of diverse educational material^51^ and the Spiderino (Jdeed et al., 2017) is offered as part of workshops to pupils in the eduLab (Pitschmann, 2019).

With all these available components on the swarm robotic research platforms, it is possible to test and evaluate swarm intelligence algorithms. This is not restricted to the software side but includes also the hardware needed to interact with the environment. This allows to draw first conclusions on the emergence of swarm behaviors and required hardware in laboratory environments. Nevertheless, the step from these research platforms to a significant number of applications or industrial products has not been achieved yet.

Nevertheless, there is already a paradigm shift in the industry. Several companies in different domains envision self-organizing solutions to the increasingly high complexity of their production plants and activities in dynamic environments. Such industry projects are, e.g., SWILT, ROBORDER, and BugWright2. This establishes an opportunity for researchers in swarm robotics and swarm intelligence to get their ideas out of the lab and into a specific application. The main issue here is to map the swarm members onto the components of an existing system and to engineer their behavior with respect to the target application. In the SWILT project, swarm members are machines, products, or lots. They must fulfill certain conditions, e.g., having computational resources to exhibit local intelligence. Nevertheless, real-world scenarios typically go beyond swarm robotics and fall into the area of swarms of cyber-physical systems. The most promising applications are in domains where it is impossible or too dangerous for humans to enter, the environment is unknown, or the real-time requirements are too restrictive to pre-compute globally optimal solutions. Specific examples could be the exploration of the deep sea, space, or celestial bodies, environmental monitoring, smart traffic concepts, or nano medicine. These visionary applications are further detailed by Schranz et al. (2020). To implement solutions in such environments, new methods, technologies, and visions are required in order to shift swarm intelligence from swarm robotic research platforms to swarm robotic products.

## 4. Conclusion

Research on swarm algorithms is a relatively young topic. Despite the large number of swarm algorithms, the transition to industry and industrial production, not to mention daily use, has not been made successfully. Nevertheless, several steps toward swarm applications have already been taken. The main objective of this paper is to motivate future research and engineering activities by providing a comprehensive list of existing platforms, projects and products as a starting point for applied research in swarm robotics.

This paper classifies basic swarm behaviors and presents a comprehensive overview of current research platforms and industrial applications. While this demonstrates the possibility of integrating basic swarm behaviors in current applications, it also shows that many applications of swarm robotics cannot fully exploit the advantages offered by distributed swarm architectures due to systems with only few agents or central control. Swarm algorithms build upon self-organized swarm behaviors, e.g., observed in natural swarm systems, such as insect colonies or flocks of birds that are able to handle extremely diverse and dynamic environments. The same holds for robot swarms. They are meant to operate in the physical world, which typically faces continual dynamic changes and must cope with events and external conditions that are hard to predict or model. Besides huge potential for applications in areas like logistics, agriculture, and inspection, one suitable working environment for swarms are places that are unsuitable for humans, including places that are hard to reach, dangerous, or dirty. Applications in these environments could help to better observe, understand and exploit the advantages of swarm behaviors: adaptability, robustness, and scalability.

In addition to industrial applications, we have also surveyed different research hardware platforms dedicated to swarm robotic experiments. On the one hand, this overview allows to choose an appropriate research platform for implementing and testing swarm algorithms in laboratory environments. On the other hand, it shows that there is a huge potential in research to transform these platforms from pure prototyping platforms to productive, industrial robotic systems that are able to perform in the real world. This might require to shift from the current simplified robot models and controls to a trade-off between simplicity of design and capability of solving complex tasks in a reliable way, e.g., from reduced resource consumption to a more intensive usage of sensor data and information sharing.

## Author Contributions

MSc: main part of research in industrial products and projects and research platforms including discussion, extension of new swarm behaviors, and overall organization of contribution and format. MSe: main part of swarm behaviors, support in research on products and projects and discussion. MU: research on swarm behaviors and adaption with new behaviors. WE: support in the discussion, abstract, and conclusion.

### Conflict of Interest

The authors declare that the research was conducted in the absence of any commercial or financial relationships that could be construed as a potential conflict of interest.

## Footnotes

^1^Balboa robot website: https://www.pololu.com/product/3575

^2^Kilobot website: http://www.kilobotics.com/

^3^K-Team website: https://www.k-team.com/

^4^Jasmine website: http://www.swarmrobot.org/

^5^Colias open source website: https://github.com/MonaRobot/Colias

^6^Colias commercial website: http://www.visomorphic.com/

^7^Mona open source website: https://github.com/MonaRobot

^8^Mona commercial website: https://ice9robotics.co.uk/

^9^Elisa-3 website: https://www.gctronic.com/doc/index.php/Elisa-3

^10^K-Team website: https://www.k-team.com/

^11^GRITSbot website: https://www.wevolver.com/wevolver.staff/gritsbot/master/blob/Overview.md

^12^Robotarium website: https://www.robotarium.gatech.edu/

^13^e-puck open source website: http://www.e-puck.org/

^14^e-puck commercial website: https://www.gctronic.com/e-puck.php

^15^Thymio website: https://www.thymio.org

^16^Pheeno website: https://discourse.ros.org/t/pheeno-a-low-cost-ros-compatible-swarm-robotic-platform/2698

^17^Spiderino website: https://spiderino.nes.aau.at

^18^Zooids website: https://github.com/ShapeLab/SwarmUI

^19^APIS website: https://github.com/wvu-irl/reu-swarm-ros (software only).

^20^Droplet website: https://code.google.com/archive/p/cu-droplet/

^21^Swarm-bots website: https://www.ercim.eu/publication/Ercim_News/enw53/nolfi.html

^22^Swarmanoid website: https://cordis.europa.eu/project/id/022888

^23^M-TRAN website: https://www.wevolver.com/wevolver.staff/m-tran

^24^ATRON website: https://www.wevolver.com/wevolver.staff/modular.atron/master/blob/Overview.md

^25^For a full list, the reader is referred to https://en.wikipedia.org/wiki/Self-reconfiguring_modular_robot

^26^Qualcomm Flight Pro website: https://www.intrinsyc.com/qualcomm-flight-pro-development-kit/

^27^DJI M100 website: https://www.dji.com/at/matrice100/info#specs

^28^bitcraze Crazyflies website: https://www.bitcraze.io/crazyflie-2-1/

^29^Optitrack website: https://optitrack.com/

^30^FINken website: https://www.ci.ovgu.de/SwarmLab/Robots/FINkens.html

^31^CORATAM website: http://biomachineslab.com/projects/control-of-aquatic-drones-for-maritime-tasks-coratam/

^32^Swarmies website: https://www.nasa.gov/content/meet-the-swarmies-robotics-answer-to-bugs

^33^Swarathon website: http://nasaswarmathon.com/

^34^SwarmFarm Robotics website: http://www.swarmfarm.com/

^35^Xaver website: https://www.fendt.com/int/xaver

^36^SWILT website: https://swilt.aau.at/

^37^SWARMIX website: http://www.swarmix.org

^38^CPSwarm website: https://www.cpswarm.eu

^39^SAGA website: http://laral.istc.cnr.it/saga/

^40^Spaxels website: http://www.spaxels.at

^41^Flyfire website: http://senseable.mit.edu/flyfire

^42^Ehang website: http://www.ehang.com

^43^Lucie micro drone website: https://veritystudios.com

^44^Apium Data Diver website: http://apium.com/data-diver/

^45^Hydromea website: http://hydromea.com/

^46^SWARMs website: http://www.swarms.eu/

^47^subCULTron website: http://www.subcultron.eu/

^48^ROBORDER website: https://roborder.eu/

^49^BugWright2 website: http://dream.georgiatech-metz.fr/?q=node/108, project start: January 2020.

^50^Sentien Robotics website: http://sentienrobotics.com/

^51^Thymio's Official Website: https://www.thymio.org/de/
